# Medical Students' Perceptions of Dementia after Participation in Poetry Workshop with People with Dementia

**DOI:** 10.1155/2016/2785105

**Published:** 2016-02-09

**Authors:** Alaina J. Garrie, Shruti Goel, Martin M. Forsberg

**Affiliations:** New Jersey Institute for Successful Aging, Rowan University School of Osteopathic Medicine, Stratford, NJ 08084, USA

## Abstract

*Purpose*. Researchers assessed whether medical students' participation in a poetry workshop with people with Alzheimer's disease and related dementias (ADRD) affected their attitudes towards persons with ADRD.* Objective*. To add to the growing body of research summarizing the impact of nonclinical interventions on medical students' perspectives about people with ADRD.* Design*. Researchers used dementia attitudes scale (DAS) and interpretive phenomenological analysis (IPA) to analyze participants' attitudes.* Setting*. Osteopathic medical school and dementia care unit in the state of New Jersey.* Participants*. Eleven out of fourteen medical students completed the study.* Measurements*. Emerging themes were classified from the postintervention semistructured interviews and descriptive statistics were used to compare the preintervention to postintervention DAS.* Results*. Researchers found statistically significant differences between preintervention and postintervention DAS scores. Study participants scored a preintervention DAS mean, 107.09 (SD = 11.85), that changed positively and significantly to the postintervention DAS mean, 121.82 (SD = 10.38). DAS subdomains, “comfort” (*P* = 0.002) and “knowledge” (*P* = 0.01), and eleven of the twenty DAS items underwent a positive and statistically significant shift from preintervention to postintervention. IPA of the interviews yielded five primary and five secondary themes, supporting the measured statistical outcomes.* Conclusion*. Medical students' participation in a poetry workshop, with people with ADRD, positively impacts their attitudes.

## 1. Introduction

The Aging, Demographics, and Memory Study (ADAMS) currently estimates that 14 percent of people aged 71 and older in the United States have dementia [1]. An estimated 5.2 million Americans have Alzheimer's disease. The number of people aged 65 and older living with Alzheimer's disease is expected to nearly triple by 2050 [2]. However, research estimates that about one-third of general practitioners find the clinical management of dementia difficult [3]. The future of dementia care lies in the hands of medical students. Yet many medical students find the complexities of older people's medical problems, including dementia, overwhelming [4, 5].

Medical trainees struggle to see persons with ADRD in a positive light [3–9]. But they lack the time to stop and question, why? [3] Bensadon et al. point to how students' aging-related attitudes negatively impact career and treatment decisions thereby contributing to the deficit of practitioners available to care for people with ADRD [6].

Experiential learning models, designed to stretch thinking modalities, without increasing time requirements, may sufficiently alter medical trainees attitudes, so they feel more comfortable communicating with and caring for people with ADRD [3, 6, 9, 10]. Studies support that artistic interventions affect the attitudes of people caring for those with ADRD [7, 9, 11–13]. A study examining the impact of a poetry intervention at one care home and one day center found that poetry helped caregivers recognize the humanness of the ADRD residents [13]. In turn, this recognition motivated the caregivers to develop positive attitudes toward people with ADRD and a desire to provide more humanistic care [11]. At a nursing home, an artistic intervention exposing medical students to the joy dementia patients derived from the activity “Time Slips,” a creative story telling program including persons with dementia, expanded participants' ability to see sufferers of ADRD in a more positive light [8]. At Columbia School of Medicine, nineteen medical students attended a single 90-minute museum-based art-centered program that engages individuals with ADRD and caregivers. After the artistic intervention at Columbia, the medical students indicated significant increases in comfort levels with ADRD patients [7]. Research supports both how attitudinal shifts impact caregiving and the use of nonclinical learning interventions for helping medical trainees see people with ADRD in a positive light.

## 2. Method

### 2.1. Participant Selection and Demographics

US News and World Report ranked Rowan University School of Osteopathic Medicine, a large medical school in the suburban Philadelphia region of Southern New Jersey, as a 2016 top program in geriatric medical education [14]. Second-year medical students take a month-long required geriatrics course which covers the physiology of aging and public health topics. The third-year medical students complete a required four-week clinical rotation in geriatric medicine at local facilities.

Rowan University School of Osteopathic Medicine medical students at all levels of training received an invitation to participate in the workshop. Researchers selected study participants by sending an email out to the first fifteen students who signed up to attend the workshop. This email informed the fifteen students about the research study and offered them an opportunity to participate in the study. Within the week prior to the workshop, researchers sent out a second email confirming that the student would participate in the workshop and the research study. The students participating in the study reviewed and signed the consent form prior to attending the workshop. Fourteen out of the fifteen workshop participants met the inclusion criteria of currently attending Rowan University School of Osteopathic Medicine.

The present pilot study only includes research data for the eleven medical students who completed all three study components: preintervention and postintervention DAS and half-hour postintervention semistructured interviews. Three students failed to complete one of the three study components. Eight first-year medical students, two third-year medical students, and one second-year student completed the preintervention and postintervention DAS and half-hour postintervention semistructured interviews. Of these students, seven identified themselves as Asian (Asian = Indian, Pakistani, Filipino, and Chinese) (63%), one participant as Black (9%), and three as White (27%). Eight out of eleven student participants were male (57%). Four out of the eleven study participants knew family members with ADRD and five out of eleven expressed an interest in geriatrics prior to attending the workshop. Researchers compared the present pilot study's demographics to that of the student body which consisted of 47.3% males, 45.4% White, 39.6% Asian, 8.3% Black, and 3.5% Hispanic out of a total of 647 students [15].

### 2.2. Setting

Medical students and assisted living facility residents with ADRD gathered at a local continuing care community, located in New Jersey, to work with Gary Glazner, the founder and Executive Director of the Alzheimer's Poetry Project, who ran the poetry workshop. Residents with ADRD, admitted to the dementia unit where the intervention took place, scored less than a twenty-four on the Mini Mental State Examination (MMSE is the accepted state-wide assessment as a part of the diagnostic protocol for dementia. A person that scores <24 on the examination receives the diagnosis of dementia in accordance with educational background). They lacked the ability to independently perform ADLs (ADL: feeding oneself, bathing, dressing, grooming, work, homemaking, and leisure) and IADLs (IADL: ability to use telephone, shopping, food preparation, laundry, mode of transportation, responsibility for own medications, housekeeping, and ability to hand finances). A board certified geriatrician evaluated and deemed each person on the unit appropriate for admission based on a physical exam, MMSE, ADL, and IADLs.

### 2.3. The Intervention

Requiring a short time commitment on the part of participants defines this intervention as a “low-intensity” learning intervention. Prior to the poetry with ADRD participants, the Rowan students attended a one-hour overview lecture of the Alzheimer's Poetry Project by Glazner at Rowan University. At the dementia unit, Glazner gave a brief, fifteen-minute introduction of his work to those in attendance. After the lecture, students and ADRD residents, led by Glazner, constructed a group poem about love. The poetry session lasted one hour. After the session, students conversed with the facility's residents. In total, the intervention lasted three and a half hours.

### 2.4. Research Design

Researchers measured preintervention attitudes with the DAS within the week of the intervention. One week following the intervention, researchers administered the postintervention DAS and conducted the postintervention, half-hour, semistructured qualitative interviews.

DAS data exists for multiple studies evaluating medical students' attitudes after participation in artistic interventions and would allow researchers to compare cross-study outcomes [7, 8]. Being a statistically valid, 20-item scale, each item of the DAS falls on a 7-point Likert scale ranging from 1 (strongly agree) to 7 (strongly disagree) [12]. The DAS holds convergent validity with the Kogan Attitudes Toward Older People Scale, Fraboni Scale of Ageism, Attitudes Towards Disabled Persons Scale, and the Interaction with Disabled Person Scale [12]. Researchers completed statistical analysis of the DAS using SPSS (version 20.0, IBM Corp., Armonk, NY).

In addition to DAS data collected in this study, researchers performed IPA of the postintervention qualitative interviews, analyzing verbatim transcripts. Two researchers administered the postintervention qualitative semistructured interviews thereby standardizing the process. The postintervention qualitative interview questions sought to discover students' perceived knowledge of dementia, general outlook of dementia as a diagnosis, and viewpoints on caring for dementia patients. Conducting half an hour postworkshop semistructured interview sessions with each participant, researchers asked participants the following questions:

*Did you attend Gary Glazner's poetry workshop? Why did you involve yourself in this activity? Did it meet your expectations? Why or why not? Do you see yourself caring for people with dementia in your medical practice? How do you think the workshop has impacted this? What are some adjectives you would use to describe someone with dementia? How do you view the quality of life of a dementia patient? Have you had high contact, low contact, or no contact with dementia patients? *

The two researchers recorded the postintervention qualitative interviews with a digital recorder with Dragon Naturally Speaking software.

The software electronically transcribed each interview and the two researchers reviewed transcription and made certain the transcripts held a verbatim accounting of students' postintervention interviews. After data collection and transcription of the interview audio, noting changes in tempo, flow of speech, intonation, and volume, a researcher trained in medical anthropological ethnographic techniques performed first-pass qualitative coding of the transcript, identifying initial themes. The researcher then went on to conduct second-pass coding critically assessing the first-pass themes and collating the coded transcripts into general theme sets. After this aggregation of second-pass themes, the researcher structured the themes into primary and secondary data sets supported with verbatim quotations. Another researcher working on the pilot study juxtaposed verbatim quotations to the primary and secondary themes ensuring accurate reporting.

Themes emerged as researchers linked the common observations vocalized by students. Quotes like that of Student 3's in which the student expressed that* “The workshop gave me more confidence I guess in not being timid or one foot back in terms of approaching people with dementia” (4.32–34)* (4 = transcript number/student number, 32–34 = line number where statement is located on transcript; researchers used transcript and line number for the multiple review of themes derived from verbatim quotes) matched peers statements. For instance, Student 3's comments echoed those of Student 10's statement:* “This experience has made me more comfortable with speaking and conversing and interaction with people that are older” (10.24)*. IPA supports the grouping of comments like those from Student 3 and Student 10 into a singular theme. The Secondary Theme 3 (the workshop increased students' confidence and comfort with approaching those with ADRD) formed from this type of analytical deconstruction of the interview transcripts. During the postintervention qualitative interview sessions, participants also filled out the postintervention DAS questionnaire.

Previous researchers of medical students' attitudes of geriatrics found that IPA delved deeper into the complexities of students' behaviors [13]. When soliciting medical students' perspectives on geriatric education using IPA at the Miller School of Medicine, researchers uncovered themes often neglected by attitude scales [16]. Public health researchers also use IPA to overcome the limitation of quantitative approaches, the failure to expand upon the lived experience of people affected by changes in healthcare policy [17]. Many researchers accept that IPA offers necessary insights for making effective educational policies with the potential to impact healthcare outcomes [17].

### 2.5. Data Handling

Researchers assigned both the questionnaires and recordings a study ID number linking to the participant and left no identifiers on either the questionnaire or the audio recording. Researchers deleted the associated key with study IDs and associated participants after the labeling of the questionnaires and audio recordings with an ID study number. Analysis of the postintervention semistructured interview transcripts occurred without access to the names of the participants.

### 2.6. Ethics

The students participated in the workshop freely, the students did not receive monetary incentives, and the intervention did not represent a course requirement although completion of the workshop would contribute to consideration for the New Jersey Institute of Successful Aging Distinguished Student Award. The protocol for this research was reviewed and approved by the Institutional Review Board of Rowan University School of Osteopathic Medicine.

## 3. Results

This arts-based, poetry workshop significantly improved medical students' attitudes towards individuals with ADRD. The DAS data (*N* = 11) showed statistically significant results and the IPA (*N* = 11) offered insight into the positive impact of the poetry intervention.

### 3.1. DAS Results

When administering the DAS, the lowest score is 20, neutral score is 80, and the highest score is 140. Eleven students completed both pre- and postintervention tests with a preintervention mean of 107.09 ± 11.85 (*t*(10) = −8.77, *P* < 0.001). After the postintervention administration, the overall DAS score changed positively and significantly to a mean of 121.82 ± 10.38 (*t*(10) = −8.77, *P* < 0.001). O'Connor and McFadden when developing the DAS found that individuals with ADRD family members may score higher at preintervention level [17].

Delving deeper into the mean DAS score data set, the present pilot study also examined whether or not DAS responses of Rowan medical students with family members affected by ADRD (4 out of 11 participants) showed significant variance from the students without family members affected by ADRD (7 out of 12 participants). Researchers conducted an independent samples test comparing overall DAS scores between participants with ADRD family members to those without ADRD relatives. The independent samples test shows that the *P* values for the overall preintervention DAS score *P* = 0.251 and overall postintervention DAS *P* = 0.707 lacked a significant difference (*t*(9) = 1.227, *P* = 0.636). These results support an equal variance between the two groups and suggest that the group with ADRD family members did not score higher than the group without ADRD family.

Table 4 summarizes the results of the paired samples* t*-tests which compared overall, subdomain, and item level DAS scores before and after the poetry workshop. Ten out of twenty items showed statistically significant differences (Table 4). Based on the paired* t*-test analysis, researchers found that the DAS items 1, 3, 4, 8, 12–16, and 19 show score improvements between the preintervention and postintervention tests using the DAS (Table 3). On the DAS, these items are grouped under the subdomains of “comfort” and “knowledge.”

Representing items from the DAS “knowledge” subdomain items, 3, 12, 14, 15, and 19 displayed statistical significance. Researchers calculated the greatest positive preintervention to postintervention change on items 14 and 19. On item 14 (people with ADRD can enjoy life), nine out of eleven participants had a positive change between preintervention and postintervention DAS, all participants showing a ≥1 point increase. On item 19 (we can do a lot now to improve the lives of people with ADRD), six out of eleven participants improved their scores between the preintervention and postintervention, all participants showing a ≥1 point increase.

The DAS items which fall under the umbrella of the “comfort” subdomain, 1, 4, 8, 13, and 16, displayed statistical significance. Item 16 (I feel frustrated because I do not know how to help people with ADRD) stood out as a comfort subdomain DAS item whose scores increased at postintervention in comparison to preintervention. Item 8 (I'm not very familiar with ADRD) showed a greater than one point increase in eight out of eleven participants' scores.

### 3.2. IPA Results

This section includes a brief discussion of several of the primary themes (Tables 1 and 2), an example highlighting a correlate between DAS data and IPA, and the voices of student responses to the intervention. The IPA of verbatim semistructured interview transcripts (*N* = 14) yielded five primary themes (Table 1) and five secondary themes (Table 2).

IPA related directly to the statistical analysis of the DAS data. For instance, in the case of DAS item 4 (I feel confident around people with ADRD) with significance (*P* < 0.019), the IPA Secondary Theme 3 (Workshop increased confidence in communicating with ADRD patients) directly supported the quantitative gains reported by DAS.

Going beyond the DAS data, after the intervention, the medical students underwent a positive shift in the way they described persons with ADRD. Students demonstrated this shift as they started articulating that those living with ADRD could enjoy themselves and express themselves creatively:

*Student 5: I guess what I viewed as what someone with severe dementia would be like was nothing I saw at the workshop. I think that one thing that the workshop definitely showed me though was that these people can be very creative, they seemed to be enjoying themselves I think to the point that I didn't even realize a lot of them had dementia  *(“a lot of them,” in reference to the ADRD of the facility's dementia unit).* It did influence me positively more so in the sense that they are still capable of a lot. (5.25–29)*

*Student 6: Honestly, when they were going around and sitting in front of him and I was waiting for someone to not be receptive. I was honestly kinda shocked that they were so receptive to him. I was just surprised that they were putting in all the input. I think it helped me see firsthand that they can have a lot of fun and enjoy themselves and almost forget like they have dementia. (6.17–20; 59-60)*

*Student 14: So you know I might have thought a certain way about them but when I was actually there interacting with them, you know everything went out the window and it's like forming a new experience and a new perspective of how they are… I thought a lot of them were very normal so. Yeah that's surprising. (14.40-8)*

Watching Glazner's work and participating through poetry encouraged students to also consider poetry as a tool for treating those with ADRD, stating the following:

*Student 9: I think the workshop helped me determine that there are other resources available to people with AD that are not just medications […] with the poetry and with the book that we got of poetry I think that I would be able to do some of that in the office if patients were willing to do it with me. So I have tools to use both in the office and with patients in between visits as well. (9.13–17)*

*Student 15: I didn't know that poetry could do such a difference […] today just increased my own knowledge that you know there's no barrier to helping people …you can use a lot of tools to make a difference. (15.12–16)*

*Student 8: Maybe hopefully even in the future it [referring to poetry] could even be part of, ah, treatment, ah, that physicians would assign to patient's family or even nursing homes. (8.33-5)*

The poetry intervention left students expressing that they now feel more comfortable interacting with people with ADRD.

*Student 10: I think I may have patients from the elderly that come to my practice and this experience has made me more comfortable with speaking and conversing with people that are older. I like don't have to stick with people my age to go out and have a good time. This would not have been done before the workshop. I feel like I could go and hang out with people with dementia, I dunno I just feel more comfortable. (10.22–26)*

Overall, the IPA and DAS analysis demonstrated significant and positive results.

## 4. Discussion

The present pilot study adds depth to the previous DAS studies which lacked the added insights of IPA. This study required a short time commitment of about 3 hours on the part of the participants but still managed to affect students' attitudes. Finding an artistic intervention with a short time commitment on the part of participants fits the hectic pace of medical students' schedules. Conducting a longitudinal study of similar interventions would reveal whether or not students' attitudes remain altered by this type of “low-intensity” intervention.

The structure of the poetry workshop in and of itself may have influenced study outcomes. Rowan University used a small grant from the Arnold P. Gold Foundation to pay for Glazner to come to the school and run the workshop. He instructed and demonstrated for participants how to employ poetry to augment the quality of life of individuals with dementia. Glazner performed “call and response” during the construction of the love poem; this encouraged participation from both the medical students and people with dementia. Other poetry preceptors might not carry out the workshop as effectively and might not produce the same effects in students' perceptions of people with ADRD [17, 18]. In addition, Mr. Glazner chose the topic of the group poem. Future research can explore what aspects of the workshop directly influence outcomes.

The Rowan participants trained in Gary's methods could theoretically carry out sessions with new sets of students and people with ADRD. Using the small group model to hold multiple workshops throughout the year, a greater number of medical students could benefit from this type of learning intervention. However, the changes attributed to the workshop might not have been as significant from students just attending the workshop. The postinterview semistructured interviews and DAS administration themselves may have also served as a therapeutic tool (self-reflection). A student remarked on the experience of filling out the DAS, stating the survey forced him to face what* “you are scared to think about, it helped me think about myself and what I thought of working with people with dementia” (2.67–69)*. The previous study with Columbia medical students that attended the museum intervention which spurred a positive change in their attitudes towards people with ADRD also filled out a preintervention and postintervention DAS [7]. Future researchers could examine whether or not an additional reflection component beyond the DAS would produce an even greater effect on students' attitudes.

Although researchers employed randomization techniques for selecting the study sample, only eleven Rowan medical students completed the study. These students' exposure to geriatrics in medical school may have also augmented the measured positive effects of the poetry workshop.* Student 14* reinforces this point when stating:* “I have participated in many, um, nursing home projects and geriatrics related seminars and I just feel a connection and I'm really interested” (14.7, 8)*. Educators working to run the poetry intervention workshops may face the difficulty of soliciting student participation. Many students view ADRD individuals and elderly in a negative light which may represent a challenge to recruitment especially in institutions without geriatric focused curriculums [12, 13, 17].

Introducing this workshop in the preclinical years may prove useful in giving medical students the tools and confidence to work with ADRD individuals by the time they reach their clinical years. In our sample, medical students at all levels of training benefited from this workshop. Ideally, medical students would get the opportunity to participate in this type of workshop each year of training.

The use of IPA and the short time frame between preintervention and postintervention administration perhaps mitigated some of the weakness associated with not using a comparison group. Studies with a larger sample size and conducted at a variety of medical institutions with various curriculums would shed even more light on the key motivating factors behind students' decisions and attitudes towards people with ADRD.

Both the IPA and DAS data offered insights into students' motivational factors for attending the workshop. Although Student 6's motivation to attend the workshop was as follows,* “My grandma has Parkinson's in the late stages, and I was just looking to get exposure to other dementia patients” (6.4-5)*, the independent samples tests performed on DAS data demonstrated that familiar connection did not influence statistical outcomes. The positive effect of the workshop superseded familiar relationships although a larger sample may change this outcome.

Researchers of the present pilot study support the use of IPA for the clarification of students' motivating factors as measured by the DAS as supported by student quotations and the primary and secondary themes (Tables 2 and 3). The results of the present pilot study support the need for workshops like the poetry intervention to help medical students to see people with ADRD outside the confines of biomedical definitions of ADRD and social media outlets' depictions of ADRD as hopeless and negative [5]. Student 10 highlights this point, when stating:

*I see someone like Meredith Gray's mother on Gray's Anatomy, the stereotype where the person is in their own mindset at some time in their life, so they are too caught up in their own world to go and pay attention to what is going on around them really. The patients I saw do not look like they are out of touch with reality. (10.53–56)  *(Meredith Gray's mother, a fictional TV character on Gray's Anatomy, has dementia)

The poetry workshop's positive influence on the students' descriptors of people with ADRD, like those of Student 10 and the overall positive change in mean DAS scores, supports the use of a poetry workshop as means to improve medical students' attitudes toward people with ADRD.

## Figures and Tables

**Table 1:** Primary themes^*∗*^ abstracted from postintervention semistructured qualitative interviews.

1	Shift in types of adjective used to describe ADRD patients: to more hope, capable of creativity, and humanness

2	Poetry intervention: a learning modality revealing the creativity and potentiality of ADRD

3	Learned how to use and consider the tool of poetry for enhancing ADRD patients' quality of life^†^

4	Surprised at the effectiveness of the nonbiomedical disease management technique

5	Expressed amazement at ADRD patients' novel and heartfelt contributions to poetry construction exercise

^*∗*^Primary theme: ≥9 out of 11 participants supported theme with verbatim quotes from transcribed semistructured interviews.

^†^10 out of 11 participants supported theme with verbatim quotes from transcribed semistructured interviews.

**Table 2:** Secondary themes^*∗*^ abstracted from postintervention semistructured qualitative interviews.

1	Medical students harbored the stereotype of ADRD patients as “agitated” prior to poetry intervention

2	Recognition that will care for elderly regardless of subspecialty

3	Workshop increased confidence and comfort with interacting with ADRD patients

4	After participating in poetry intervention consider using as a prescribed therapeutic modality

5	Saw the poetry intervention as a fun, happy, and positive experience way to learn

^*∗*^Secondary theme: ≥4 out of 11 participants supported theme with verbatim quotes from transcribed semistructured interview.

**Table 3:** Primary theme 1: adjectival changes.

Transcript number	Preintervention adjectives	Postintervention adjectives
1	“Quiet, reserved, frightened, moody, hyper focused, and kind” (33)^*∗*^	“Kind, lively, happy, and funny” (38)

2	“*Hopeless*, trivial-like the management, to be honest waste of space and resources” (32)	“Definitely ***not hopeless***, that's it” (33)

4	“Lost and afraid,” (29)	“*Hopeful* and trying,” (20, 31)

5	“Dependent” (30); “Agitated” (33-34)	“Friendliness” (43); “It seemed like they *could enjoy life *and you know interact” (49, 50)

7	“Trapped, sad” (33)	“*Potential*, sad,* hopeful*” (34)

8	“Crazy, bipolar, sometimes, completely normal” (81–83)	“Still part of them that's there. *Human beings*. Self part is still there for the some of those patients” (89–91)

9	“Lively in some ways, creative” (24-25)	“More so lively and creative” (26)

10	“Cant think of any before” (42)	“After APP, umm, I would use the adjective creative, funny[…]**human** because they still express the same emotions as everyone else[…] not robots or degenerate vegetable or anything like that but […]alive or something like that” (43–46)

15	No adjective given before intervention	“I would say cheerful and full of life and sincere and just funny” (41–43)

14	“Frustrated I guess, annoyed by people” (53-4)	“Really appreciate like people trying to help them out. It was just the opposite, I just can't find the real words to describe it” (58–60)

^*∗*^Numbers following quotes refer to line in interview transcript where statement is found.

**Table 4:** DAS descriptive statistics of student participants in poetry workshop (*N* = 11).

DAS items	Preintervention mean ± SD	Postintervention mean ± SD	*P* value (≤0.05)
Item 1: It is rewarding to work with people who have ADRD	5.54 ± 1.03	6.27 ± 0.90	0.012
Item 2: I am afraid of people with ADRD	6.27 ± 1.01	6.73 ± 0.65	0.180
Item 3: People with ADRD can be creative	5.18 ± 1.54	6.55 ± 0.69	0.016
Item 4: I feel confident around people with ADRD	5.27 ± 1.19	6.27 ± 0.90	0.019
Item 5: I am comfortable touching people with ADRD	5.18 ± 1.72	6.09 ± 0.70	0.074
Item 6: I feel uncomfortable being around people with ADRD	6.18 ± 1.08	6.18 ± 1.53	1.000
Item 7: Every person with ADRD has different needs	6.27 ± 1.10	6.18 ± 0.98	0.780
Item 8: I am not very familiar with ADRD	5.09 ± 1.58	6.00 ± 1.34	0.002
Item 9: I would avoid an agitated person with ADRD	3.82 ± 1.33	4.64 ± 1.36	0.068
Item 10: People with ADRD like having familiar things nearby	5.82 ± 1.17	5.91 ± 1.64	0.867
Item 11: It is important to know the past history of people with ADRD	6.27 ± 0.90	6.18 ± 1.25	0.724
Item 12: It is possible to enjoy interacting with people with ADRD	5.91 ± 1.22	6.72 ± 0.47	0.020
Item 13: I feel relaxed around people with ADRD	4.91 ± 1.14	6.27 ± 1.01	0.013
Item 14: People with ADRD can enjoy life	5.00 ± 1.61	6.45 ± 0.52	0.005
Item 15: People with ADRD can feel when others are kind to them	6.09 ± 1.04	7.00 ± 0.00	0.016
Item 16: I feel frustrated because I do not know how to help people with ADRD	3.64 ± 1.36	4.72 ± 2.15	0.025
Item 17: I cannot imagine taking care of someone with ADRD	4.45 ± 1.92	5.45 ± 1.86	0.110
Item 18: I admire the coping skills of people with ADRD	5.27 ± 1.56	5.73 ± 1.27	0.210
Item 19: We can do a lot now to improve the lives of people with ADRD	5.36 ± 1.63	6.36 ± 0.67	0.049
Item 20: Difficult behaviors may be a form of communication for people with ADRD	5.55 ± 1.03	6.09 ± 0.94	0.140
DAS total	107.09 ± 11.85	121.81 ± 10.38	<0.001
Comfort subdomain (items 1, 2, 4, 5, 6, 8, 9, 13, 16, and 17)	50.36 ± 8.49	58.63 ± 7.66	0.002
Knowledge subdomain (items 3, 7, 10, 11, 12, 14, 15, 18, 19, and 20)	56.72 ± 7.95	63.18 ± 4.81	0.010
